# Chemistry

**Published:** 1884-10

**Authors:** Wm. H. Taggart

**Affiliations:** Freeport, Ill.


### CHEMISTRY.

BY WM. H. TAGGART, D. D. S., FREEPORT, ILL.

Read before the Illinois State Dental Society.

Possibly there is no branch of the natural sciences that has more of the mysterious connected with it than chemistry. The various objects which constitute external nature present to the observer an infinite variety of quality and circumstances. Some are brittle; others are elastic and tough. Some natural objects are endowed with life; others are lifeless; they may be moved, but do not move themselves. Some bodies are in a state of incessant change; while others are so immovable and unchangeable that they seem to be everlasting. In the midst of this diversity of external objects, where lies the domain of chemistry?

When air moves in wind, when water moves in tides, or in the fall of rain or snow, the air and the water remain air and water still; their constitution is not changed by the motion, however frequent or however great. A bit of granite thrown off from the ledge by frost, is still a bit of granite, and no new or altered thing. If a solid bit of iron be reduced to filings, each finest morsel is metallic iron still, of the same substance as the original piece, as will appear if a piece be sufficiently magnified under the microscope. The melted fluid lead in the hot crucible, and the solid lead of the cold bullet cast from it, are the same in subtance, only differing in respect to temperature. In all these cases the changes are external and non-essential; not intimate and constitutional. They are called physical changes, and do not change the composition of the molecules, and, therefore, do not change the nature of the substance acted upon.

When iron is exposed to the weather it becomes covered with a brownish coating, which bears no resemblance to the original iron, and if exposed long enough, the metal completely disappears, being changed into this very different substance, rust. So, too, the fragment of granite broken from the ledge, exposed for centuries to the action of air and rain, becomes changed, and after a time could not be recognized as granite. All these changes involve alterations in the intimate constitution of the bodies which undergo them; they are called "chemical changes," and do affect the nature of the substance.

To show, in a single experiment, a physical and a chemical change, I have made a solution of calcium chloride in water, which makes simply a physical change; I pour with it a small quantity of sulphuric acid, another physical change, for it simply divides the acid; but the moment the two substances unite, we have a complete change in the nature of the substance, or chemical change, and, as you see, the two transparent fluids are changed into a white solid, and have formed a substance in which you can neither recognize calcium chloride or sulphuric acid.

Mixtures of two or more substances may be formed by mingling them in all conceivable proportions, but a compound formed by chemical action consists of certain invariable proportions of its constituents. Thus, oxygen and hydrogen may be mixed in any desired proportions, but they will unite to form water only in the ratio of one part oxygen to two parts hydrogen. When iron rusts, the oxygen of the air combines with the metal at the rate of three ounces of oxygen to seven ounces of iron. No chemist can make three ounces of oxygen unite with six ounces of iron. In a mixture the constituents are said to be free; in a compound they are said to be in combination.

Possibly a greater proportion of our success as dentists, in treating the cases as presented to us, comes from thorough cleanliness. Another large item of our success lies in the cleanliness of our offices and persons, and as water is the type of cleanliness, it may not be out of place to show the composition of this most abundant and useful article; at the same time this will show you what is meant by the chemical symbols. In the first place, we will take the water, and ask of what it is composed. In this case I shall use electricity as the key to open it up. By means of electricity the chemist is enabled to decompose almost every known compound. I have here an ordinary bichromate potash battery, the poles of which are connected with this vessel containing water, with a few drops of acid added to make it a better conductor of electricity; over the poles of this battery I place two test tubes, to collect what is given off. [Experiment.] You see that one pole has generated twice as much gas as the other. Now, if I take this larger volume of gas and place a lighted match in it, the light is immediately extinguished, showing that the gas will not support combustion, but it takes fire and burns itself, showing that it is different from air. [Experiment.] This gas has been named hydrogen. The other gas, if treated the same way, not only supports combustion, but increases the flame, showing it to be different from air and the other gas H, and one volume only was present to two volumes of the H, showing the composition of water to be $H_2O$. This decomposition was produced by what is called "electrolysis." Now, if we can form water again out of these two gases, it will be conclusive proof that this is the composition of water. If these two gases were mixed together again and ignited, we would have a violent chemical combination followed by a loud report, and a drop of water would be the result. This can be shown in a crude way by burning H in the air as in this experiment. I form the H gas and light the small flame, and over this hold a glass tube; the musical tone made is caused by minute explosions as the hydrogen unites with the oxygen of the air, and you see the tube is covered with moisture, and a drop of water forms.

As water is composed of $H_2O$, it is a very easy transition to come to that much talked of compound peroxide of hydrogen, which has for a symbol $H_2O_2$, or one more atom of O, or in other words, oxygenated water. This substance in the pure state is a syrupy liquid, having a disagreeable metallic taste, somewhat resembling tartar emetic. When taken into the mouth it causes a tingling sensation, increases the flow of saliva, and bleaches the tissues with which it comes in contact. It is a very unstable compound, and even at ordinary temperatures is decomposed. The dilute substance, as we use it, is, however, more stable, and can be boiled, and even distilled, without suffering decomposition. Substances containing sulphur are decomposed by it at the expense of half its O. Its therapeutical action in the treatment of alveolar abscess has been explained in this way: "When the peroxide of hydrogen comes in contact with pus, the extra O it contains is liberated so rapidly that the hydrogen and sulphur of the tissues immediately combine with it, resulting in $H_2SO_4$, or sulphuric acid in small quantity, sufficient to glaze the surface of the pus-producing area, thus affording an opportunity for the exuding protoplasmic material to organize into new tissue. The remaining unsatisfied atoms of O quickly distend the pus sac, and force the contents through the root or fistulous opening."—(*Harlan.*)

To show that these chemical changes do take place, I have made some sulphureted hydrogen ($H_2S$), the principal disagreeable substance found in pus or in the decomposition of albuminous substances, and to this I add the peroxide of hydrogen $H_2O_2$, and test the product formed, and find it to be sulphuric acid. [Experiment.]

While we are on this subject of remedies for alveolar abscess, it may be a very good time to speak of carbolic acid, our old stand-by. This substance is made from coal tar, and is a more agreeable antiseptic than creosote. That there is a chemical difference between them can be easily shown, and for those who are anxious to know for certain which they are using (as the one is so often sold for the other), the test can be made in a few minutes:

*First*—Carbolic acid is soluble in glycerine; creosote is not.

*Second*—Carbolic acid precipitates nitro-cellulose from collodion; creosote does not.

*Third*—Carbolic acid gives a brown color with ferric chloride and alcohol; creosote gives a green color with ferric chloride and alcohol.

*Fourth*—Carbolic acid gives a violet color with ferric chloride and water; creosote gives a green color with ferric chloride and water.

Carbolic acid coagulates albumen, and as albumen is one of the principal ingredients of pus, it is a question whether the wholesale use of carbolic acid in these cases is admissible, especially when the pus is forming. Just the fact that the case gets along well by using it, is not always proof positive that the proper medicinal remedy has been used; for the same case, treated with a remedy that would not coagulate the pus, might have got along much better, and there would not have been the insoluble compound formed for nature to remove by absorption; in other words, the simple pus would be easier to absorb than the insoluble coagulated pus. Let those who have implicit faith in the action of these remedies (carbolic acid and creosote) in all cases, try treating their next case of alveolar abscess by simply going through the same process with water, and they will be surprised at their success, and will come to the conclusion that success in the treatment of alveolar abscess comes in a great measure from persistent *mechanical* cleanliness.

In using either of these two remedies in the cases mentioned, the object is said to be to disinfect the tooth. Now, a disinfectant is a smell-destroyer, and these two substances are disinfectants only in a very small degree, and that small degree is due more to the mechanical removal of the decomposed mass than to any chemical combination caused by the remedies. To show that carbolic acid is not a true disinfectant, we have simply to place it in contact with the most disagreeable odor we have to remove, which is sulphureted hydrogen. I have here some water saturated with this offensive gas; now, by adding the carbolic acid, even in excess, we have no diminution of the smell, simply for the reason there has been no chemical change. If I now add chloride of zinc to the same kind of a solution, a chemical change takes place, and the smell is destroyed, showing the proper action of a disinfectant. [Experiment.]

To show this chemical change I form the equation $H^2S+ZnCl^2=ZnS+2\ HCl$. In this change the chlorine, which always has a great affinity for hydrogen, has united with it, and thus broken up the compound, destroyed the smell, and formed hydrochloric acid, 2 HCl.

There are a great many in the profession who cry down amalgam fillings, not because they think they will not save teeth, but because they say that the mercury they contain has an injurious effect on the health of the patient. For the benefit of those who take this ground, and the fact that it comes legitimately under the head of chemistry, I have here a very delicate test for the presence of mercury, either free or in combination with metals, as we find it in amalgam. By taking a solution of nitrate silver and adding ammonia until it becomes cloudy, and then adding enough more to make a clear solution, and using this as an ink to write with, and placing the paper over the amalgam plug and allowing it to remain in the dark the writing turns dark, showing that the mercury is constantly giving off fumes. I have performed this experiment with an amalgam plug that was in a tooth 42 years, and it apparently gives the test as well as a younger one, and the beauty of it all is that the patient's health was not destroyed.

Another of our common remedies, that has a few interesting chemical thoughts connected with it, is arsenic. This is a remedy that does not lose its identity very readily; in other words, it remains as arsenic, I suppose, for an indefinite period, and may be detected as a poison months and even years after it has been used. I have here a solution made from the pulp of a tooth destroyed by arsenic; the remedy was allowed to remain in the tooth two weeks. I have very carefully followed the rule for detecting arsenic by Marsh's test; that is, by testing the purity of the zinc and acid to find that no arsenic was present to begin with. Now, by pouring the solution containing the pulp into the bottle where nascent hydrogen has access to it, and passing it through chloride of calcium to absorb all moisture, and by burning the gas, we have formed on a piece of cold porcelain held in the flame a characteristic black sooty deposit. [Experiment.] This is a very delicate test and accurate, for we cannot get this same kind of a spot from any other substance that we can use. It is said that the one seven-thousandth of a grain can be detected. This test was performed with the small end of the pulp, showing that the arsenic was absorbed into the pulp and was not merely on the surface, as some say. To show the destructive effects which arsenic has, and to show what might come from the careless use of this remedy, I have here a piece of the process from around the roots of a lower molar, the death of which was caused by the careless use of arsenic. It seems the patient, a young lad sixteen years old, went to a dentist to have an aching tooth extracted.

The dentist pulled the crown off, leaving the pulp entirely exposed and the roots still in their place; of course it ached fearfully, and the boy was afraid to have it touched with the forceps again, so the dentist said he would destroy the nerve by putting medicine on it, so he put arsenic directly on the exposed pulp and bleeding gum without any precaution whatever. In the course of a few days the face commenced to swell and the patient fell into my hands, and a worse gangrenous looking mass could hardly be imagined than that presented by looking into his mouth. In the course of a week the gum had all sloughed off that side of the jaw, and the process around the broken tooth and the adjacent ones began to come away in pieces. This piece I show you was the largest one, and was the process from around the broken tooth. Immediately after the patient came into my hands I took away as much of the diseased parts as I could get, and made the test as just shown, and found arsenic. This shows conclusively that the remedy used was arsenic, and it also shows the results that may follow its careless use.

It is a well-known fact that the manufacturers of the zinc preparations seem to persist in sending out twice as much powder as is necessary for the liquid. For the benefit of those who wish to utilize their extra powder, I think it proper to tell how to make the oxyphosphates. The liquid is phosphoric acid. This is made by the action of sulphuric acid on old bones or by burning phosphorus in oxygen. The kind used for the cement preparations is called glacial phosphoric acid, and can be bought at any drug store (it is a solid), and by adding sufficient water to dissolve it, and evaporating this until it is of the consistency of glycerine, we have an excellent liquid that will go with any of the powders. The powder is made from oxide zinc bought at the drug store, and by calcining it at a white heat for two hours and then grinding it in a mortar we have an excellent powder. Some manufacturers put in borax, silica, and powdered glass before calcining it: and the addition of these ingredients and the difference in the burning is what makes one cement work differently from another.

